# Expression and Localization of Neuregulin-1 (Nrg1) and ErbB2/ErbB4 Receptors in Main Endocrine Organs of the Rhesus Monkey

**DOI:** 10.5812/ijem.9871

**Published:** 2013-07-01

**Authors:** Wei-jiang Zhao

**Affiliations:** 1 Center for Neuroscience, Shantou University Medical College, Shantou, People's Republic of China

**Keywords:** Neuregulin-1, ErbB2, ErbB4, Rhesus Monkey, Pituitary, Thyroid, Parathyroid, Adrenal Gland

## Abstract

**Background:**

Although Neuregulin-1 （Nrg1）and its receptors have been indicated at the mRNA level in partial human endocrine organs and its functional roles have been evaluated in vitro, their morphological distribution in higher animals are not fully studied. The present research focused on expression of Nrg1 and its main receptors ErbB2 and ErbB4 in main endocrine organs of the rhesus monkey.

**Materials and Methods:**

The morphological expression of Nrg1 and its receptors ErbB2 and ErbB4 as well as their potential co-localization were determined by double immunofluorescence in the pituitary, thyroid, parathyroid, pancreas and adrenal gland sample tissues. The expression level of Nrg1 on each sample was indexed by the fold of integrative fluorescence intensity (IFI) relative to that of one cortical tissue.

**Results:**

Differential expression of Nrg1 and their cognate receptors ErbB2 and ErbB4 were found selectively expressed in endocrine organs we tested, with higher expression levels detected in the adrenal gland (AG) and pancreas. Co-localization of Nrg1 with either ErbB2 or ErbB4 was detected in AG, thyroid and parathyroid gland, and Nrg1 was only co-localized with ErbB4 in the islet cells of the pancreas. In the pituitary, adjacent localization of Nrg1 positive cells with ErbB4 positive cells were observed.

**Conclusions:**

This investigation morphologically profiles the differential expression of Nrg1 and its receptors ErbB2 and ErbB4 in the main endocrine organ structures, suggesting an autocrine or paracrine-directed Nrg1-ErbB signaling pathway in some of these structures.

## 1. Background

Neuregulin-1 (Nrg1) is one of the most active members of the epidermal growth factor (EGF)-like family (1). At leastsix isoforms of Nrg1, including type I to III Nrg1α and Nrg1β, due to the alternative splicing of Nrg1 gene, have been identified (2). Interaction of Nrg1 with the dimers of its receptors, including ErbB2, ErbB3 and ErbB4, results in many biological processes (3-6). Nrg1 receptors were reported to be expressed in hypothalamic astrocytes, where their activation as a result of paracrine Nrg1 stimulation, is essential for stimulating secretion of luteinising hormone-releasing hormone, as well as for puberty (7, 8). Recently, Nrg1 was also detected in gonadotroph cells of the anterior pituitary, where it is assumed to mediate prolactin secretion from the lactotrophs in a juxtacrine manner (4, 6). In addition, the interaction of Nrg1 with ErbB3 receptor has been reported to induce prolactin (PRL) secretion from the rat somatolactotroph GH3 cells (5, 9). An extensive distribution of Nrg1 in the anterior pituitary was observed at Estrous 1 (E1) and E2 phases accompanied by apparent phosphorylated activation of ErbB4 in rats (6).

In the juvenile and adult rhesus monkeys widespread expression of ErbB2, ErbB3 and ErbB4 receptor mRNAs throughout the telencephalon were reported (10). In addition, Nrg1 was therapeutically applied in experimental heart failure in the rhesus monkey, and led to a positive therapeutic effect by impairing rapid pacing-induced apoptosis and increasing activity of PKB and Bcl-xl proteins (11-13). In contrast, no morphological expression of Nrg1 and its receptors was systematically described in main endocrine organs of the rhesus monkey.

## 2. Objectives

We thus aimed at investigating the morphological expression of Nrg1 and its specific ErbB2 and ErbB4 receptors in main endocrine organs: anterior pituitary, thyroid, parathyroid, adrenal gland (AG) and pancreas. These findings may further our understanding of the Nrg1/ErbB receptor signaling-based functions in the endocrine organs.

## 3. Materials and Methods

### 3.1. Reagents and Microarray 

Mouse anti-Nrg1α /β antibodies were purchased from Lab Vision (Fremont, CA, USA). Rabbit anti-ErbB2 and ErbB4 antibodies were purchased from Beijing Biosynthesis Biotechnology (Beijing, China). Donkey anti-mouse secondary antibody conjugated to DylightTM 488 and donkey anti-rabbit secondary antibody conjugated to DylightTM 594 were purchased from Jackson Laboratory (Jackson Labs, Bar Harbor, Maine, USA). The rhesus monkey organ tissue microarrays were obtained from Chaoying Biotechnology (RhFDA1, Xi’an, Shaanxi, China).

### 3.2. Immunofluorescence Staining

The rhesus monkey organ tissue microarray sections (4 μm thick and deparaffinized) were rehydrated through a graded series of ethanol to PBS. Antigen retrieval was performed using 10 mM citrate buffer (pH 6.0). The samples were blocked with 10% normal donkey serum in PBS at room temperature for 60 min. The samples were incubated overnight at 4°C with the following primary antibodies: mixture of mouse monoclonal anti-Nrg1 α/β (1:100; Ab-1 7D5, Lab Vision, Fremont, CA, USA) and rabbit anti-ErbB2 or rabbit anti-ErbB4 antibodies (1:100; Bioss , Beijing China). After having been washed in PBS three times for 5 min each, the samples were incubated at room temperature with donkey anti-mouse secondary antibody conjugated to DylightTM 488 (1:500) and donkey anti-rabbit secondary antibody conjugated to DylightTM 594 (1:1000) for 90 min. Nuclei were counterstained with DAPI. Images were acquired using a Zeiss Fluorescence Microscopy system (AXIO IMAGER Z1, Zeiss, Germany). DAPI, DylightTM 488 and DylightTM 594 were excited at 405 nm, 488 and 594 nm, respectively.

### 3.3. Integrated Immunofluorescence Intensity (IFI)

Integrated fluorescence intensity (IFI) was used to evaluate the protein levels of Nrg1α/β in the organ tissues. The IFI at each tissue point was obtained using the MultiImageTM Light Cabinet CY3 filter of the FluorChem HD2 gel imaging system (Alpha Innotech, CA, USA) for Nrg1α/β , and the optical density was analyzed using Image Tool II software (University of Texas Health Science Center, San Antonio, TX, USA). The IFI was evaluated on the basis of a gray scale ranging from 0-255, and was expressed as the fold increase over that of the brain cortical tissue.

### 3.4. Statistical Analysis

The statistical analyses were performed using SPSS (Statistical Package for the Social Sciences) 10.0 software (SPSS, Chicago, IL, USA). The data were analyzed using the Student’s t-test for independent samples. P values < 0.05 were considered statistically significant.

## 4. Results

### 4.1. IFI of Nrg1 in Different Endocrine Organs

**Figure 1:**
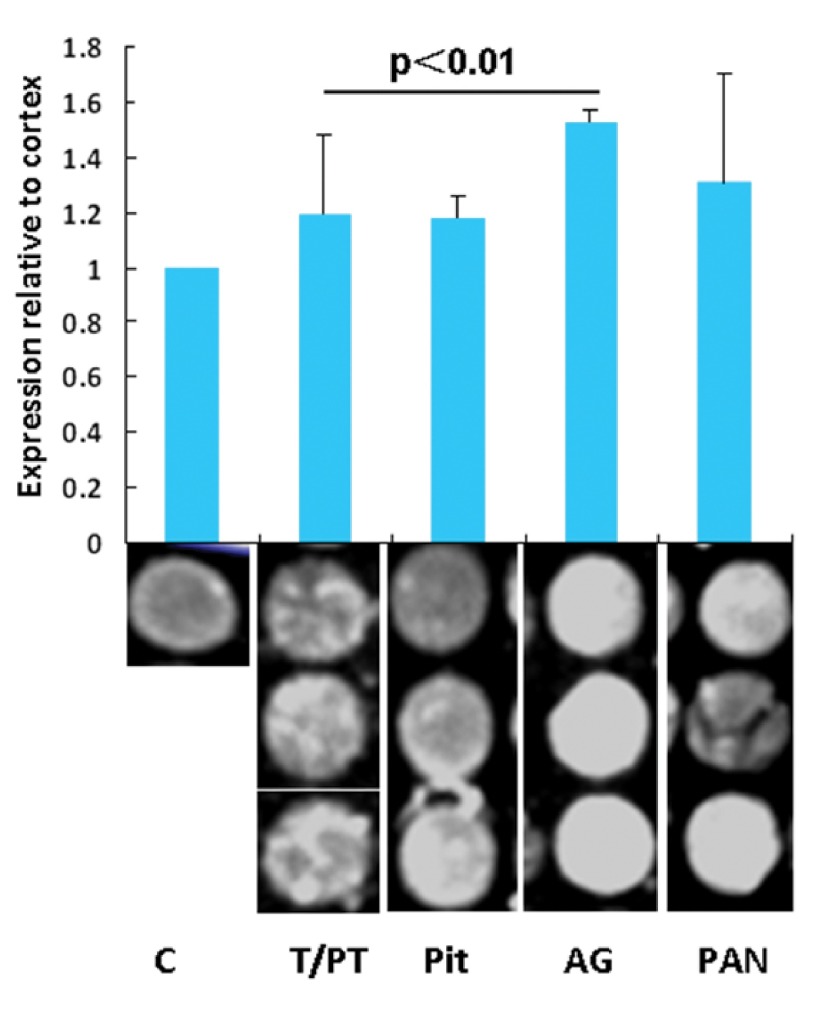
Fold of Integrated Fluorescence Intensity (IFI) of Neuregulin-1 (Nrg1) Relative to the Cortex in the Endocrine Organs of the Rhesus Monkey. Shown are mean values ± standard deviation from 3 tissue points for each organ.

 To evaluate the levels of Nrg1 expression in different endocrine organs, the IFIs for each tissue point were analyzed. Relative expression was determined by comparing the optic intensity with that of the randomly selected cortex as a base line control. Nrg1 expression relative to that of the brain cortex in the pituitary, thyroid/parathyroid, AG, and pancreas tissue samples are 1.19 ± 0.29, 1.18 ± 0.08, 1.52 ± 0.05, and 1.31 ± 0.39, respectively. The Nrg1 level relative to the brain cortex from the AG is significantly higher than that in the thyroid (P ＜ 0.01). No significance was found among other organs in Nrg1 levels (Figure 1).

### 4.2. Expression and Localization of Nrg1 and ErbB2/ErbB4 in the Anterior Pituitary of Male Rhesus Monkey

**Figure 2:**
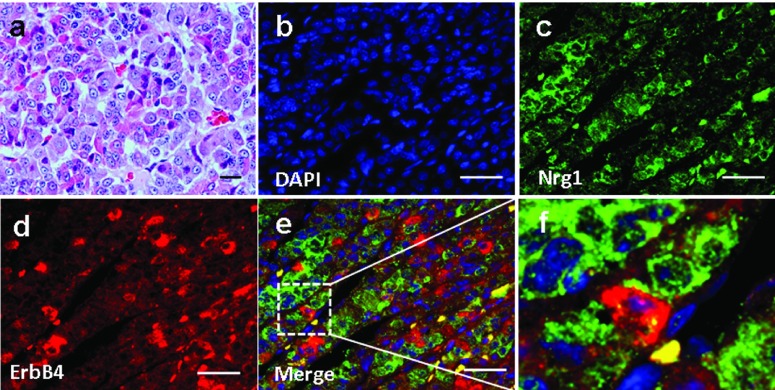
Expression and Localization of Nrg1, as Well as ErbB2/ErbB4 in the Anterior Pituitary of the Rhesus Monkey. (a) Haematoxylin-eosin (H & E) staining. (b-e) Double Immunofluorescence Staining for Nrg1 and ErbB-4 were revealed in the pituitary of the rhesus monkey. Blue, DAPI stained nuclei. Adjacent staining of Nrg1 and ErbB4 were revealed in the pituitary (inset). Scale bars = 50μm.

 We investigated Nrg1 and ErbB4 localization in the anterior pituitary of 3 male Rhesus monkeys aged 5-7 years. Basic pituitary structures based on HE staining was shown in Figure 2a. The expression of Nrg1 and ErbB4 was observed, which showed a partial adjacent pattern, suggesting the existence of an Nrg1/ErbB4 juxtacrine signaling in the anterior pituitary in non-human primates (Figure 2 b-f). In contrast, ErbB2 was not detected (data not shown).

### 4.3. Expression and Localization of Nrg1 and ErbB2/ErbB4 in the Thyroid/Parathyroid Tissue

**Figure 3.**
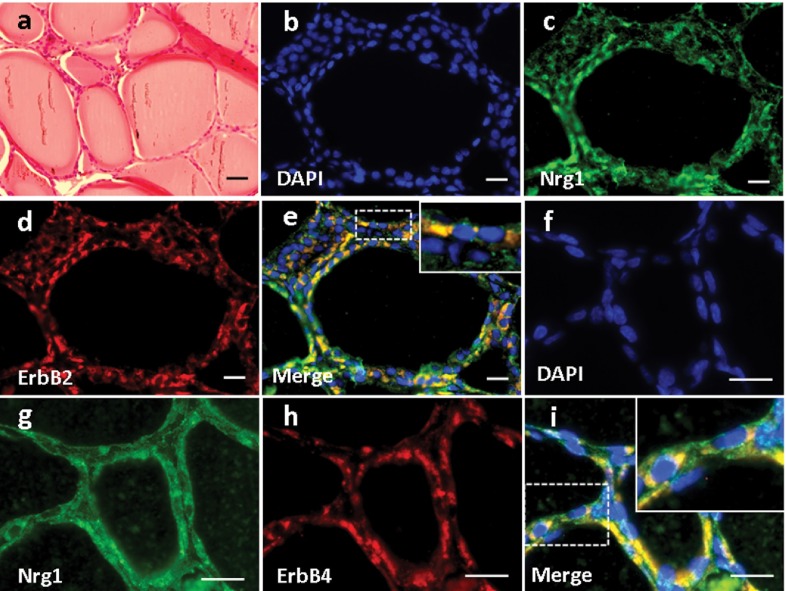
Expression and Localization of Nrg1, as well as ErbB2/ErbB4 in the Thyroid of the Rhesus Monkey. (a) H & E staining. (b-i) Double Immunofluorescence Staining for Nrg1 with either ErbB2 (b-e) or ErbB4 (f-i) was shown. Scale bars = 50μm.

High magnification of H&E-stained thyroid tissue was shown in Figure 3a. Based on immunofluorescence staining, we found that Nrg1 was expressed in both the follicular and parafollicular cells (with larger cell body but lighter stained nuclei) of the thyroid gland (Figure 3 e, g), where it co-localized with both ErbB2 (Figure 3 b-e) and ErbB4 (Figure 3f-i).

**Figure 4:**
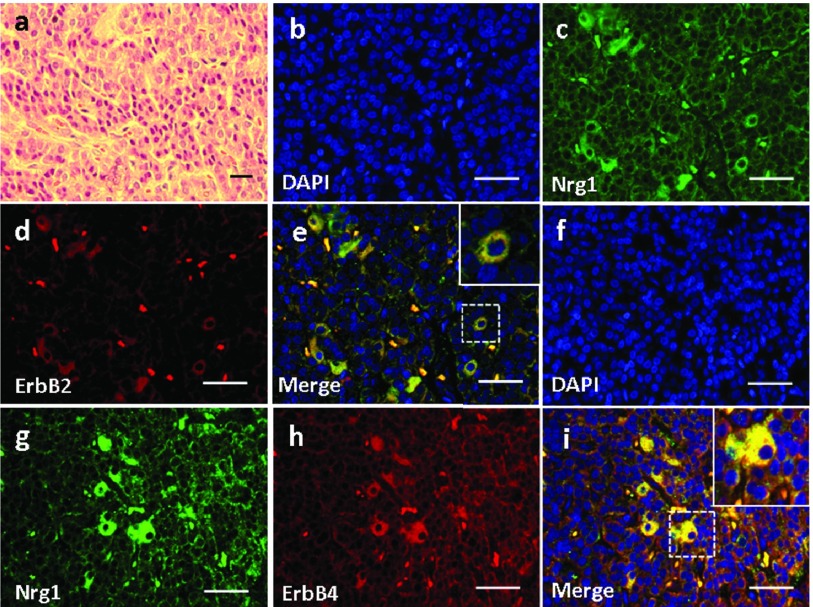
Expression and Localization of Nrg1, as well as ErbB2/ErbB4 in the Parathyroid of the Rhesus Monkey. (a) H & E staining. (b-i) Double Immunofluorescence Staining for Nrg1 with either ErbB2 (b-e) or ErbB4 (f-i) was shown. Scale bars = 50μm.

Basic parathyroid structures based on HE staining was demonstrated in Figure 4a. The larger polygonal oxyphil cells, which constitute a smaller population, showed positive staining of Nrg1 (Figure 4e, g). In addition, co-localization of Nrg1 with both ErbB2 (Figure 4b-e) and ErbB4 (Figure 4f-i) was found mainly in these cells.

### 4.4. Expression and Localization of Nrg1 and ErbB2/ErbB4 in the Adrenal Gland (AG) Structure

**Figure 5.**
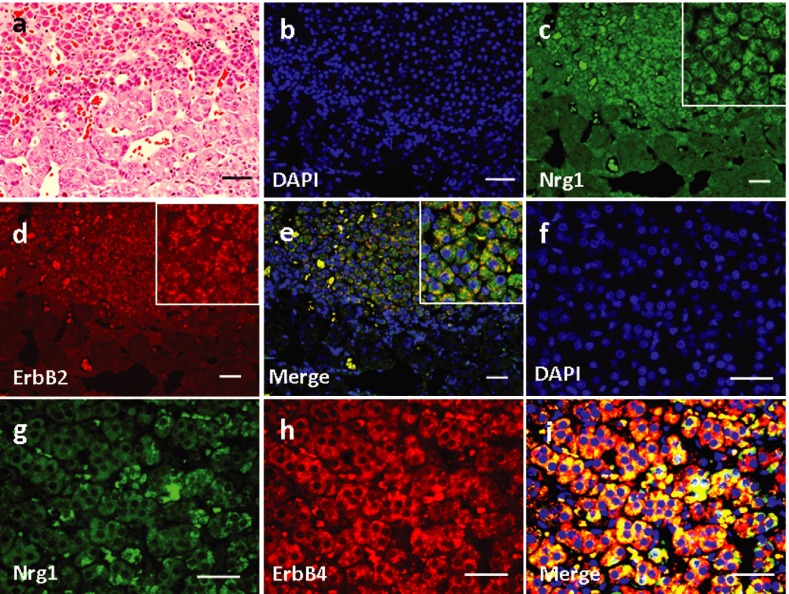
Expression and Localization of Nrg1, as well as ErbB2/ErbB4 in the Adrenal Gland (AG) of the Rhesus Monkey. (a) H & E staining. (b-i) Double Immunofluorescence Staining for Nrg1 with either ErbB2 (b-e) or ErbB4 (f-i) was shown. Scale bars = 50μm.

Basic structures of AG based on H&E staining was demonstrated in Figure 5a. In the AG, Nrg1 was detected in the zona reticularis (Figure 5c, g), in which it co-localized with either ErbB2 (Figure 5b-e) or ErbB4 (Figure 5f-i). In contrast, neither ligand nor receptors were observed in the zona medullaris (Figure 5b-i).

### 4.5. Expression and Localization of Nrg1 and ErbB2/ErbB4 in the Pancreas

**Figure 6.**
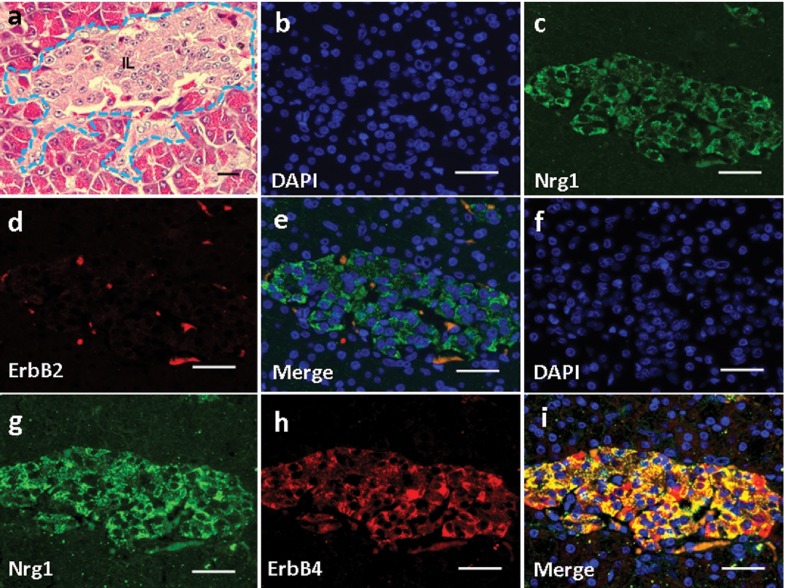
Expression and Localization of Nrg1 as well as ErbB2/ErbB4 in the Pancreas of the Rhesus Monkey. (a) H & E staining. (b-i) Double Immunofluorescence Staining for Nrg1 with either ErbB2 (b-e) or ErbB4 (f-i) was shown. IL, islet. Scale bars = 50μm.

Basic pancreatic structures based on HE staining was shown in Figure 6a. In the pancreas, Nrg1 was solely detected in the islet cells (Figure 6c, g). ErbB2 was not detected in any cell types in the pancreas (Figure 6d), and no co-localization of Nrg1 with ErbB2 was observed (Figure 6b-e). In contrast, ErbB4 was detected in the pancreatic islet cells (Figure 6h), where the co-localization of Nrg1 with ErbB4 was observed (Figure 6f-i).

## 5. Discussion

To profile the expression and localization of Nrg1 and its receptors in main endocrine organs in non-human primates, we immunofluorescently co-stained Nrg1 with either ErbB2 or ErbB4 in the pituitary, thyroid/parathyroid, AG, and the pancreas. In the anterior pituitary, the finding that Nrg1 positive cells are localized adjacently with cells expressing ErbB4 suggests the existence of a juxtacrine interaction between Nrg1 and ErbB4. This was in contrast to that in the pituitary of the female Wistrar-Furth rats, where Nrg1 is co-localized with ErbB4 (6). 

Nrg1 expression has never been explored and ErbB oncogene encoded receptors have not been detected in normal adrenal gland tissues to our knowledge. However, amplifications and deletions of the ErbB oncogenes was frequently detected in human pheochromocytoma, a human endocrine neoplasms derived from the adrenal gland (14). The co-localization of Nrg1-ErbB4 co-localization suggested the possible interaction of Nrg1 with its cognate receptor ErbB4 in the function of pancreas islet cells. However, the interaction of ErbB4 with other EGF-like lignads cannot be excluded, including ErbB4 interaction with betacellulin (BTC). NRG-4 has been reported to regulate the lineage determination of islet cells during pancreatic development, as well as to increase DNA synthesis in rat insulinoma cells (15-17). ErbB4 mRNA level also correlates with pancreatic cancer with lymph node and distant metastases (18). However, the sole expression of ErbB4 in the pancreatic islet was of total contrast to other reports showing predominant presence in the ductal and acinar cells but with a lower level in islet cells. Work by Huotaris (16) suggests that ligands of the EGF-R/ErbB-1 and ErbB4 receptors regulate the lineage determination of islet cells during pancreatic development. Betacellulin （BTC） also acts through EGF-R/ErbB-1 to induce the differentiation of beta-cells. Hyperplasia of the pancreas in response to Nrg1β1 was also observed in mice (19). It has been reported that Nrg1 and ErbB receptors expression declines by birth and resumes early in postnatal life in mice. Our findings about Nrg1/ErbB receptors expression may suggest that these molecules might be relevant to islet development and regrowth in high primates (20).

In conclusion, we demonstrated differential Nrg1 expression pattern and co-localization pattern of Nrg1 with either ErbB2 or ErbB4 in main endocrine organs in the rhesus monkey, suggesting distinct roles of Nrg1 mediated by ErbB2 and/or ErB4 receptors in different endocrine organs. Their roles may possibly be achieved through the paracrine pattern, the autorine and juxtacrine patterns. Thus, in vitro investigation using tumor cell lines treated with Nrg1 may provide more insight into its roles in endocrine organ specific functions.
